# Single-port cholecystectomy in a patient with *situs inversus totalis *presenting with cholelithiasis: a case report

**DOI:** 10.1186/1752-1947-6-96

**Published:** 2012-04-03

**Authors:** Marcus Vinicius Dantas de Campos Martins, José Luis Pantaleão Falcão, James Skinovsky, Guilherme Moraes Silva Simões de Faria

**Affiliations:** 1 Hospital Municipal Lourenço Jorge, Av. Ayrton Senna 2000, Barra da Tijuca, Rio de Janeiro, Brasil; 2 Universidade Estácio de Sá. Rua Riachuelo 27, Lapa, Rio de Janeiro, Brasil; 3 Universidade Positivo/Hospital Universitário Cruz Vermelha, Av. Vicente Machado 1310, Curitiba, Brasil

## Abstract

**Introduction:**

*Situs inversus totalis *(mirror image organs) is a rare condition and may affect the intra-abdominal viscera as well as the intrathoracic organs. Cholelithiasis is not more common in these conditions, but the diagnosis may be more difficult.

**Case presentation:**

We present the case of a 59-year-old African woman with gallstones and *situs inversus totalis*. A single-port cholecystectomy was performed using a single trocar access device (SITRACC).

**Conclusions:**

The procedure was uneventful, showing that this approach may be an option for this kind of surgery even in patients with *situs inversus totalis*.

## Introduction

*Situs inversus totalis *(SIT; the 'mirror image' reversal of major organs) is a rare congenital abnormality that affects approximately 0.005% of all live births [1]. There have been several reports of laparoscopic cholecystectomy for cholelithiasis in *situs inversus totalis*, none of them using a single-port device [2-6].

Our group has been working on the development of single-port platforms and natural orifices surgeries since 2006 [7]. We published our first experimental study in 2008 [8] and the Anvisa (Brazilian National Health Surveillance Agency) approved this device, called SITRACC (for "single trocar access'), for human use in 2009. In the same year we coordinated and published a multicenter study of SITRACC cholecystectomies that included 81 cases [9].

A comprehensive search of the PubMed, Scielo and Embase databases was performed in August of 2011 using medical subject headings 'cholecystectomy', 'cholecystectomy, laparoscopic' and 'situs inversus'. There is a published report on single-incision multiport laparoscopic cholecystectomy for a patient with situs inversus totalis [10], but there is no data published regarding a single-port technique.

## Case presentation

A 59-year-old African woman presented to our surgical clinic with a few months history of intermittent left upper quadrant pain aggravated by fatty food. Imaging via an ultrasound scan showed abdominal *situs inversus *with gallstones in a left-sided gallbladder. A pre-operative chest X-ray showed dextrocardia with *situs inversus *with no evidence of bronchiectasis.

**Figure 1:**
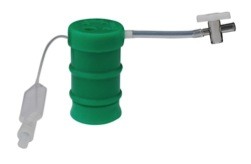
**The single trocar access device (SITRACC)**.

**Figure 2:**
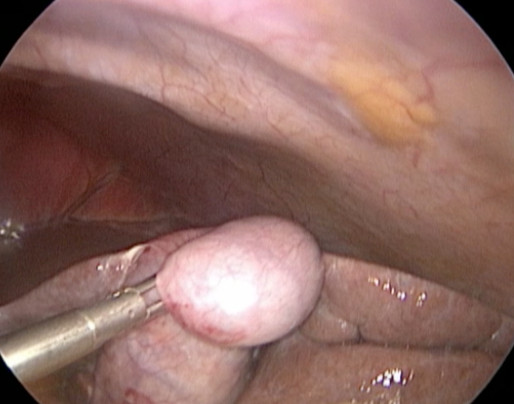
**Gallbladder retraction**.

**Figure 3:**
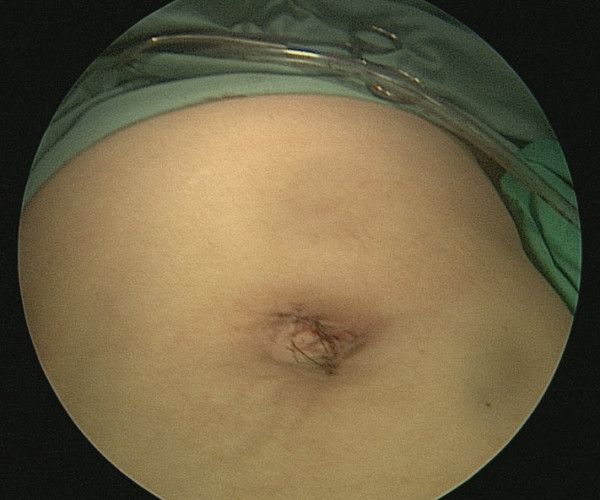
**Single-port incision closed**.

A single-port cholecystectomy was performed using a SITRACC device (Figure 1). Our patient was placed in the supine position with both the surgeon and camera operator on his right side and the assistant on the left side. The monitor was placed near our patient's head at the left side. The SITRACC device was placed using an open technique. A flexible grasper retracted the gallbladder (Figure 2). A Calot dissection was performed using a left-hand grasper and hook. Clips were applied to both cystic artery and duct. The gallbladder dissection from the liver was uneventful. The specimen was delivered through the umbilicus and the fascia and skin were closed (Figure 3). Our patient had an uneventful post-operative course and was discharged on the second post-operative day.

## Discussion

*Situs inversus totalis *may affect the intra-abdominal viscera as well as the intrathoracic organs [2]. It can be associated with many other anatomical variations, including heart malformations and Kartagener's syndrome (bronchiectasis and sinusitis coexistence) [2,3]. There is no evidence to suggest that gallstones are more common in people with this condition. However, left upper quadrant pain may delay the diagnosis. An ultrasound scan can confirm the presence of gallstones and the left-sided gallbladder. Some authors suggest that it would be very useful to perform a magnetic resonance cholangio-pancreatography (MRCP) procedure in order to reveal the exact anatomy of the biliary tract [3], thus decreasing intra-operative complications and enabling better planning of the surgical procedure. Pre-operative knowledge of the presence of malrotation and its details is of prime importance, even more so when performing laparoscopic or minimally invasive surgery.

Since a great variety of patterns of malrotation have been described, the pre-operative knowledge of the presence of malrotation is of prime importance because the surgeon can then plan the operation better and minimize the possibility of intra-operative complications, especially when performing laparoscopic or minimally invasive surgery [10].

In 1991, Campos and Sipes were the first to report a successful laparoscopic cholecystectomy in a patient with *situs inversus *totalis [7]. Since then, many other cases have been published [2-5], even including cases of acute cholecystitis. Some technical difficulties, such as the mirror image, for example, should be considered. It requires the surgeon to stand on the right side with the monitor above the patient's left shoulder. This disposition requires mental adaptability and manual dexterity. The orientation and ergonomic challenges may result in an increased operative time.

In recent years, interest in performing new minimally invasive approaches has increased worldwide. Some reports have been published for abdominal access performed only through the umbilicus. This technique was named single port access (SPA) surgery. In a meeting held in July of 2008, the Single Port Consensus reviewed all the terminology for laparoscopic or endoscopic procedures performed through a single incision in the abdomen and proposed that laparoendoscopic single-site surgery (LESS) be used as the common term to define this procedure. LESS techniques basically include two different types of surgery. In the first one a single incision is made to place multiple trocars. In the second, a single incision is made to place a single trocar designed to contain all instruments. There are many models of single-port devices from many industries. SITRACC (Edlo, Porto Alegre, Brazil), Tri-port (Advanced Surgical Concepts, Wicklow, Ireland), X-Cone (Karl Storz, Tuttlingen, Germany) and SILS (Covidien, Mansfield, USA) are some of them.

Han *et al. *first published a single-incision multiport laparoscopic cholecystectomy in a patient with *situs inversus totalis *in 2011 [11] but there are no data published regarding the single-port technique.

## Conclusions

Laparoscopic cholecystectomy is the treatment of choice for symptomatic cholelithiasis. This approach should be the same for patients with *situs inversus totalis*. LESS is a safe method that offers at least an aesthetic advantage over the traditional laparoscopy. Additional studies are needed to confirm other advantages such as less pain and less metabolic response.

## Consent

Written informed consent was obtained from the patient for publication of this manuscript and any accompanying images. A copy of the written consent is available for review by the Editor-in-Chief of this journal.

## Competing interests

The authors declare that they have no competing interests.

## Authors' contributions

MVDdCM was the surgeon and the major contributor to writing the manuscript. JLPF was the first assistant. GMSSdF was the second assistant. JS helped with the instruments. All authors reviewed the manuscript
